# Characteristics of patients dying within 30 days of diagnosis of breast or colorectal cancer in Scotland, 2003–2007

**DOI:** 10.1038/sj.bjc.6606036

**Published:** 2011-01-04

**Authors:** D H Brewster, D I Clark, D L Stockton, A J Munro, R J C Steele

**Affiliations:** 1 Information Services Division, NHS National Services Scotland, Gyle Square, 1 South Gyle Crescent, Edinburgh EH12 9EB, UK; 2 Department of Surgery and Molecular Oncology, Ninewells Hospital and Medical School, Dundee DD1 9SY, UK

**Keywords:** breast neoplasms, colorectal neoplasms, co-morbidity, mortality, Scotland, survival

## Abstract

**Background:**

Recent research has shown that most of the excess risk of death following breast and colorectal cancer in England compared with Norway and Sweden occurs in older age groups during the first year, and especially in the first month of follow-up. The aim of this study was to explore the characteristics of patients dying within 30 days of being diagnosed with one of these cancers in Scotland during 2003–2007.

**Methods:**

Anonymised cancer registry records linked to hospital discharge and death records were extracted. The study population was divided into patients who died within 30 days of diagnosis (cases) and those who survived beyond this threshold (controls). Differences in patient-, tumour-, and health service-related characteristics were assessed using the *χ*^2^-test and logistic regression.

**Results:**

Patients dying within 30 days were more likely to be elderly and to have experienced emergency admission to non-surgical specialities. Their tumours were less likely to have been verified microscopically, but they appeared more likely to be of high grade and advanced in stage. A substantial number of patients died from causes other than their cancer.

**Conclusion:**

These results suggest that early mortality after a diagnosis of breast or colorectal cancer may be partly due to comorbidity and lifestyle factors, as well as due to more advanced disease. Further research is required to determine the precise explanation for these findings and, in particular, if any potentially avoidable factors such as delays in presentation, referral, or diagnosis exist.

Survival from major epithelial cancers appears to be lower in the United Kingdom than in the Nordic countries, with the interesting exception of Denmark (Sant *et al*, 2009). More detailed analyses suggest that most of the excess risk of mortality in the United Kingdom compared with the Nordic countries occurs within a year of diagnosis (Engholm *et al*, 2007; Thomson and Forman, 2009; Holmberg *et al*, 2010) and particularly within a month of diagnosis in older patients with breast cancer (Møller *et al*, 2010) or colorectal cancer (H Møller, personal communication). A popular interpretation, which underpins the National Awareness and Early Diagnosis Initiative in England (Department of Health, 2007), is that delays in presentation and/or referral, resulting in more advanced disease stage at diagnosis, are responsible for much of the survival deficit observed in patients with cancer in the United Kingdom. However, there are alternative explanations for the excess risk of early death. These include the following: increased comorbidity leading to increased operative risks; smoking, obesity, alcohol abuse, and other factors related to lifestyle, which might both increase surgical risks and limit options for non-surgical therapies; and socioeconomic deprivation, which has been associated with increased risk of early death, although the mediators of any such effect are undoubtedly complex.

The aim of this study was to examine the characteristics of patients with breast or colorectal cancer who die within 30 days of diagnosis, using health service data collected routinely and, using proxy indicators, to explore the influence of socioeconomic deprivation, co-morbidity, and lifestyle on outcome.

## Materials and methods

Anonymised records relating to patients diagnosed with breast cancer (ICD-10 C50) or colorectal cancer (C18-C20) during the period 2003–2007 were extracted from the Scottish Cancer Registry (SCR). Patients registered on the basis of a death certificate only were excluded (<1% of cases for both cancer sites). Only the first breast or colorectal cancer arising in an individual was included in the analysis.

Patient-, tumour-, and health service-related variables from the SCR were supplemented with variables derived from a permanently linked database of acute hospital discharge records, cancer registrations, and mortality records (Kendrick and Clarke, 1993). These supplementary variables included the following: any hospital admission with chronic obstructive pulmonary disease (COPD) in the 10 years preceding diagnosis of breast/colorectal cancer as a very crude proxy for smoking history; any hospital admission with alcohol-related diagnoses in the 10 years preceding diagnosis of breast/colorectal cancer; the number of hospital in-patient bed days in the period of 10 years up to 1 year preceding diagnosis of breast/colorectal cancer (as a crude indicator of general preexisting comorbidity); the number of emergency admissions to hospital in the 30 days either before or after diagnosis of breast/colorectal cancer; and place of death (from the full mortality record) for deceased patients. The diagnostic codes used to identify COPD and alcohol-related diagnoses are shown in Appendix 1. On the basis of the postcode of residence at the time of diagnosis, patients were assigned to a deprivation fifth using the Scottish Index of Multiple Deprivation (Scottish Government, 2006). This has seven domains (income, employment, education, housing, health, crime, and geographical access) at datazone level, which have been combined into an overall index to identify area concentrations of multiple deprivation.

Mode of presentation was investigated by examining the SCR ‘method of first detection’ variable, and the type of admission, speciality, and main diagnosis leading to hospital admission in the first hospital episode of the continuous in-patient stay, during which time the cancer was first mentioned on a hospital discharge record (restricted to admissions for the cancer occurring within 30 days of either side of the SCR incidence date).

The study population was divided into patients who died within 30 days of incidence/diagnosis (cases) and those who survived beyond this threshold (controls). Follow-up was until the end of 2008.

For each characteristic examined, the *χ*^2^-test of association was used to assess differences in proportions between cases and controls. Logistic regression modelling was used to generate adjusted odds ratios and their 95% confidence intervals. Cause of death and place of death were not included as factors in the logistic regression model because, by definition, the period of follow-up differed between cases and controls.

## Results

The study included 36 484 patients. The distribution of early deaths, later deaths, and percentage of the cohort alive at the end of follow-up according to cancer site is summarised in Table 1. The proportion of early deaths was significantly higher in patients with colorectal cancer (6.6%) compared with patients with breast cancer (1.3% *P*<0.001). The difference in the percentage of each cohort alive at the end of follow-up (50.3% for colorectal cancer; 79.3% for breast cancer) was also statistically significant (*P*<0.001).

### Breast cancer

The distribution of characteristics of cases and controls is shown in Table 2. On the basis of univariate analysis, patients dying within 30 days of diagnosis were significantly more likely to be elderly; to lie outside the least-deprived category; to spend more time in hospital in the 10 years before diagnosis; to have been previously admitted for COPD; to have had one or more emergency admissions in the 30 days around diagnosis; to have had their tumour diagnosed without histology; and to have received no active treatment within 30 days of diagnosis. A lower rate of microscopic verification resulted in higher proportions of pathology-derived variables with unknown values among cases, making interpretation difficult. However, there is some evidence that cases were more likely to present with advanced disease, and less likely to present with low-grade tumours or oestrogen receptor-positive tumours. Just under 66% of cases had their primary cause of death attributed to breast cancer, and just over 70% of cases died in hospital.

The results of logistic regression modelling are shown in Table 3. Interactions were considered between age with other covariates, and stage with other covariates, and only inclusion of an interaction between age and the emergency admission variable resulted in a better-fitting model. After adjustment for covariates, the following factors remained predictive of death within 30 days of diagnosis: age group, deprivation category, emergency admissions, microscopic verification status, tumour stage, tumour grade, and absence of any recorded treatment within 30 days of diagnosis.

Of the 249 patients dying within 30 days of diagnosis of breast cancer, the ‘method of first detection’ was recorded as incidental finding in 47 (18.9%) cases, clinical presentation with relevant symptoms or signs in 153 (61.4%) cases, incidental finding at autopsy, previously unsuspected in 6 (2.4%) cases, ‘other’ in 19 (7.6%) cases, and not known in 24 (9.6%) cases. No cases were recorded as screen detected or interval cancers.

Only 155 (62.2%) cases and 11 268 (58.8%) controls had a hospital discharge record mentioning breast cancer with admission occurring within 30 days of the SCR incidence date. First admission to these continuous in-patient stays was recorded as emergency in 134 (86.5%) cases compared with 866 (7.7%) controls. Only 44 (28.4%) of these 155 cases were first admitted under general surgery, compared with 9916 (88%) of 11 268 controls. A total of 87 (56.1%) cases were first admitted under general medicine, a related subspeciality, or medicine for the elderly. Breast cancer was recorded as the primary diagnosis (main condition) in 91 (58.7%) cases compared with 10 635 (94.4%) controls.

### Colorectal cancer

The distribution of characteristics of cases and controls is shown in Table 4. On the basis of univariate analysis, patients dying within 30 days of diagnosis were significantly more likely to be elderly; to lie outside the least-deprived category; to have spent more time in hospital in the 10 years before diagnosis; to have been previously admitted for COPD; to have had previous alcohol-related admissions; to have had one or more emergency admissions in the 30 days around diagnosis; to have had their tumour diagnosed without histology; and to have received no active treatment within 30 days of diagnosis. As for breast cancer, a lower rate of microscopic verification resulted in higher proportions of pathology-derived variables with unknown values among the cases, making interpretation difficult. However, there is some evidence that cases were more likely to present with advanced disease and possibly less likely to present with low-grade tumours. Cases were less likely to have tumours of the rectum or rectosigmoid, although around a quarter of them did not have a tumour subsite specified. The male/female ratio was 1.2 among controls, but 1.0 among cases. Just under 73% of cases had their primary cause of death attributed to colorectal cancer, and just over 82% of cases died in hospital.

The results of logistic regression modelling are shown in Table 5. Interactions were considered between age with other covariates and Dukes' stage with other covariates. Inclusion of interactions for age and the emergency admission variable, age and microscopic verification status, age and any treatment variable, and Dukes' stage and treatment resulted in a better-fitting model. After adjustment for covariates, the following factors remained predictive of death within 30 days of diagnosis: age group, deprivation category, previous in-patient bed days, previous admissions for COPD, previous alcohol-related admissions, emergency admissions, microscopic verification status, tumour subsite, Dukes' stage, tumour grade, and treatment within 30 days of diagnosis. The relationship between treatment and outcome was complex: stage A, B, or C cases were more likely to have received treatment within 30 days than were controls, whereas stage ‘D’ or unstaged cases were less likely to have received treatment.

Of the 1129 patients dying within 30 days of diagnosis of colorectal cancer, the ‘method of first detection’ was recorded as screening examination in one (0.1%) case, incidental finding in 22 (1.9%) cases, clinical presentation with relevant symptoms or signs in 937 (83.0%) cases, incidental finding at autopsy, previously unsuspected in 20 (1.8%) cases, ‘other’ in 61 (5.4%) cases, and not known in 88 (7.8%) cases. No cases were recorded as interval cancers.

Only 858 (76.0%) cases and 10 609 (66.5%) controls had a hospital discharge record mentioning colorectal cancer, with admission occurring within 30 days of the SCR incidence date. First admission to these continuous in-patient stays was recorded as emergency in 685 (79.8%) cases compared with 3881 (36.6%) controls. Only 495 (57.7%) of these 858 cases were first admitted under general surgery, compared with 7536 (71.0%) of 10609 controls. A total of 276 (32.2%) cases were first admitted under general medicine, a related subspeciality, or medicine for the elderly. Colorectal cancer was recorded as the primary diagnosis (main condition) in 620 (72.3%) cases compared with 9401 (88.6%) controls.

## Discussion

The main strengths of our study are the large number of patients included (over 36 000) and the availability of information for a geographically defined population with universal health care and complete coverage by cancer registration. The study should include the same unselected spectrum of patients included in successive iterations of the EUROCARE survival studies. Identifying the population dying within 30 days is crucially dependent on the definition of diagnosis or incidence date. Unlike many cancer registries that choose from a hierarchy of dates dependent on the timing of microscopic verification, the SCR applies a definition published previously by the International Agency for Research on Cancer (MacLennan, 1991). Essentially, this allocates the earliest available date from a list of options, irrespective of microscopic verification status. In practice, this is usually the date of first relevant hospital contact for the cancer.

At first sight, the relatively low proportion of cases and controls with relevant hospital discharge records occurring within 30 days of the cancer registry incidence date seems surprising. However, there are a number of potential explanations for this. For example, in the case of elective referrals, the first relevant hospital contact (and, therefore, the source of the cancer registry incidence date) is likely to be an outpatient consultation – in some cases, the time to in-patient admission may have exceeded 30 days. In some cases, if patients are discharged from hospital before a pathology report is issued, the definitive diagnosis of cancer may not be recorded. The overall accuracy of main diagnosis on hospital discharge records is estimated to be around 88% (Information Services Division, 2007); it follows that the diagnosis will inevitably be misclassified and/or miscoded in a proportion of cases.

The weaknesses of our study reflect the problems associated with relying on routinely collected data: for example, problems of attribution (of cause of death); problems of information completeness (lack of information on stage or histological grade for some tumours); and problems of surrogacy (the need to use diagnosis of COPD as a proxy for smoking status, or the use of number of hospital in-patient bed days as a proxy for comorbidity). In brief, we have obtained statistical power and representativeness at the expense of finer clinical details. The main weakness of our study is the absence of information on any delays in presentation, referral, or diagnosis, as well as direct information on lifestyle risk factors. As a result, we are unable to determine whether patients dying early have advanced disease because of delays in presentation, referral, or diagnosis, or because of more aggressive disease, or both, and if there seem to be any avoidable factors contributing to their death. These questions can probably only be addressed by further research involving review of medical records. Indeed, at least some of the patients will already fall within the scope of the existing and well-established Scottish Audit of Surgical Mortality (Thompson and Stonebridge, 2005).

There are three main reasons for patients dying within a few weeks of the diagnosis of cancer: advanced, biologically aggressive disease by the time of diagnosis – death is directly due to the cancer itself; death due to complications related to treatment of the cancer – death is indirectly related to the cancer; and death due to some coincidental cause – death is not related to the cancer, either directly or indirectly. Any analysis of early deaths after the diagnosis of cancer has specifically to consider which of these reasons might apply. Earlier diagnosis of cancer might help prevent deaths in the first and second categories but would be unlikely to have any effect on the rate of intercurrent deaths. Conversely, comorbidity would have an impact on the second and third categories, but have limited impact on the first.

As expected, the overall mortality rate was higher in patients with colorectal cancer and the rate of early death was five times higher in patients with colorectal cancer. These findings reflect the differences in anatomy and the role of surgery in the two different types of tumour. Emergency surgery for breast cancer is an extreme rarity, whereas 20% of operations for colorectal cancer are classed as urgent or emergency procedures (Tekkis *et al*, 2003). Operative mortality is much higher for colorectal cancer than it is for breast cancer – around 5% for elective colorectal surgery, up to 20% for emergency colorectal surgery (Tekkis *et al*, 2003), and less than 1% for breast cancer surgery (El-Tamer *et al*, 2007). These factors will account for the differences noted when Tables 2 and 4 are compared: only 23% of patients with breast cancer who died within 30 days of diagnosis had received any form of treatment for their tumour; the corresponding figure for patients with colorectal cancer was 38%.

Unsurprisingly, multivariable analysis suggests that, compared with patients surviving beyond 30 days, patients with breast cancer dying early were more likely to be elderly; less likely to be affluent; more likely to have been admitted as an emergency within 30 days of diagnosis; less likely to have had their tumours microscopically verified; more likely to have advanced disease (or stage not known); more likely to have high-grade tumours (or grade not known); and less likely to have had any treatment within 30 days of diagnosis. Of the patients with breast cancer dying within 30 days, just over 34% were recorded as having an underlying cause of death other than breast cancer. Patients dying early from colorectal cancer had similar characteristics but in addition were more likely to have had a previous admission for COPD, and more likely to have had a previous alcohol-related admission. In contrast to patients with breast cancer, they were more likely to have had treatment within 30 days of diagnosis, at least for disease stages A–C, perhaps reflecting the fact that they had presented acutely with complications of their tumour, such as obstruction or peritonitis, and required urgent surgery. However, it may be unwise to draw definite conclusions about the relationship between treatment and outcome from these observational data. Of the patients with colorectal cancer dying within 30 days, just over 27% were recorded as having an underlying cause of death other than colorectal cancer. Patients dying early after a diagnosis of breast or colorectal cancer were less likely to follow a conventional pathway to diagnosis – for example, their first relevant admission to hospital was less likely to be under the care of a surgeon.

Our ‘control’ group included some patients who will have survived just a few days longer than our ‘cases’. Had we been able to compare the characteristics of patients dying early with a selected group of longer-term survivors, the differences that we observed would most likely have been accentuated considerably.

The limited impact of socioeconomic position on early deaths was unexpected, but may reflect limitations of the area-based Scottish Index of Multiple Deprivation as an indicator of individual status, and the fact that a high proportion of early deaths occurred in the very elderly, in an age range less commonly attained by people from deprived communities in which life expectancy is lower than average. Similarly, failure to show a clear effect of previous in-patient bed days may reflect better-than-average health of individuals who survive beyond the age of 85 years, as well as inclusion of related covariates, such as previous admissions for COPD, previous alcohol-related admissions, and emergency admissions in the model.

There is certainly evidence from other studies that some patients present themselves for the first time many weeks or months after the onset of relevant symptoms, and some of the reasons for prehospital delays have been identified (Macleod *et al*, 2009). Awareness of cancer warning signs appears to be low in Britain, particularly in those who are males, younger, and from lower socioeconomic status groups or ethnic minorities, and there are a number of perceived barriers for consulting (Robb *et al*, 2009). There is some amount of evidence that interventions delivered to individuals may increase cancer awareness and that interventions delivered to communities may promote awareness and early presentation (Austoker *et al*, 2009). However, it is of course also important to monitor potential adverse effects of any interventions, such as overwhelming diagnostic services.

At the same time, evidence that delays are associated with more advanced disease stage or lower survival is inconsistent and sometimes counterintuitive. It seems that some patients with aggressive, poor prognosis disease are more likely to present quickly and to be fast-tracked by health services. In fact, the paradox of patients with prolonged duration of symptoms, but who have a better prognosis than patients with shorter duration of symptomatic disease, was highlighted many years ago (Feinstein, 1964). Even then, it was recognised that some patients with longer clinical histories probably had tumours that were biologically less aggressive.

Unfortunately, studies on the impact of delay on outcome suffer from a number of limitations, including differing definitions of delays, differing ways of measuring delays, failure to take account of aggressiveness of tumours, failure to allow for emergency admissions (particularly relevant for colon cancer), and failure to take account of lead-time bias (Neal, 2009). Thus, although there may be inevitability about presentation with advanced disease for some individuals, this should not be used to defend avoidable delays faced by other patients living with the anxiety that they may (or do) have cancer.

One further avenue of research that might yield insights is the impact of lifestyle factors on survival from (as opposed to risk of) cancer. The calculation of relative survival can allow for competing causes of death associated with lifestyle factors, but cannot adjust fully for direct effects of these factors on cancer prognosis. For some cancers, there is evidence that smoking is associated with more advanced stage of disease at diagnosis, although it is not clear whether this reflects a difference in health-seeking behaviour or a direct effect of tobacco on tumour biology (Longnecker *et al*, 1989; Kobrinsky *et al*, 2003). Furthermore, there is evidence that smoking and some other lifestyle factors reduce the effectiveness of a range of treatments, including surgery, radiotherapy, and chemotherapy (Gritz *et al*, 2005; Gritz and Demark-Wahnefried, 2009), and smoking has been shown to have an independently adverse impact on cause-specific survival in patients who have had resections for colorectal cancer (Munro *et al*, 2006). We have been unable to find data on the prevalence of continuous active smoking among patients with cancer in different countries; however, whereas estimates of overall adult smoking prevalence are considerably lower in Sweden than in Scotland (Scottish Public Health Observatory, 2010), estimates for other Nordic countries are more similar to Scotland, suggesting that smoking is unlikely to be the sole explanation for the reported survival deficit in Scotland. For operable patients, there is evidence that both smoking and alcohol abuse increase the risk of postoperative complications, and there is also some evidence that intensive presurgical interventions aimed at addressing these risk factors can improve outcomes (Tønnesen *et al*, 2009). Obesity and lack of physical activity also seem likely to increase the risks associated with surgery and tackling these problems in the interval between diagnosis and surgery might have a role in reducing the risk of early death. There is also emerging evidence that personality type, mental well-being, and resilience may influence outcomes, and these factors may also be important influences on early mortality (Aspinwall and MacNamara, 2005; Mackenbach, 2010).

In conclusion, we have shown that patients dying within 30 days of a diagnosis of breast or colorectal cancer have many unfavourable prognostic characteristics. Further research is required to determine the mechanisms through which these effects are mediated and, more particularly, whether interventions aimed at dealing with them can improve survival.

## Figures and Tables

**Table 1:** Distribution of early deaths, later deaths, and percentage of the cohort alive at the end of follow-up, stratified by cancer site

				**Survived beyond 30 days**			
**Cancer site**	**All**	**Died within 30 days**	**%**	**Total**	**Alive**	**Dead**	**% of cohort alive at the end of 2008**
Breast	19 409	249	1.3	19 160	15 382	3778	79.3
Colorectal	17 075	1129	6.6	15 946	8582	7364	50.3

**Table 2:** Characteristics of patients dying within 30 days of diagnosis of breast cancer in Scotland; period of diagnosis 2003–2007

	**Cases**		**Controls**		
**Characteristic**	**Number**	**%**	**Number**	**%**	***P*-value**
*Age group*					<0.001
<65	35	14.1	10 676	55.7	
65–74	49	19.7	4212	22.0	
75–79	41	16.5	1614	8.4	
80–84	48	19.3	1374	7.2	
85+	76	30.5	1284	6.7	
					
*SIMD fifth* a					0.013
1-Least deprived	28	11.2	3887	20.3	
2	55	22.1	4008	20.9	
3	60	24.1	4023	21.0	
4	57	22.9	3880	20.3	
5-Most deprived	49	19.7	3358	17.5	
					
*Previous inpatient bed days*					<0.001
0	111	44.6	7771	40.6	
1–7	48	19.3	6431	33.6	
8–28	40	16.1	3408	17.8	
29+	50	20.1	1550	8.1	
					
*Previous admission for COPD*					<0.001
Yes	19	7.6	462	2.4	
No	230	92.4	18 698	97.6	
					
*Previous alcohol-related admission*					0.157
Yes	8	3.2	369	1.9	
No	241	96.8	18 791	98.1	
					
*Emergency admissions within 30 days of diagnosis date*	<0.001				
0	77	30.9	17 732	92.5	
⩾1	172	69.1	1428	7.5	
					
*Microscopically verified*					<0.001
Yes	134	53.8	18 943	98.9	
No	103	41.4	196	1.0	
Not known	12	4.8	21	0.1	
					
*Tumour stage*					<0.001^b^
I	14	5.6	6710	35.0	
II	10	4.0	7783	40.6	
III	8	3.2	1506	7.9	
IV	56	22.5	648	3.4	
Not known	161	64.7	2513	13.1	
					
*Tumour grade*					<0.001^c^
I	3	1.2	2343	12.2	
II	23	9.2	7536	39.3	
III	23	9.2	6441	33.6	
Not known	200	80.3	2840	14.8	
					
*ER status*					<0.001^d^
Positive	73	29.3	14 668	76.6	
Negative	22	8.8	3024	15.8	
Not known	154	61.8	1468	7.7	
					
*Any treatment within 30 days of diagnosis*	<0.001				
Yes	57	22.9	12 880	67.2	
No	192	77.1	6280	32.8	
					
*Underlying cause of death*					0.092
Breast cancer	163	65.5	2350	62.2	
Other cancer	10	4.0	298	7.9	
Diseases of circulatory system	40	16.1	572	15.1	
Diseases of respiratory system	15	6.0	158	4.2	
Any other cause	21	8.4	400	10.6	
					
*Place of death* e					<0.001
NHS acute hospital	179	71.9	1951	51.8	
Home	37	14.9	746	19.8	
Hospice	7	2.8	477	12.7	
Other institution	26	10.4	594	15.8	

Abbreviations: COPD=chronic obstructive pulmonary disease; ER=oestrogen receptor; SIMD=Scottish index of multiple deprivation.

a SIMD not known for four controls.

b *P*<0.001 excluding not known category.

c *P*=0.222 excluding not known category.

d *P*=0.118 excluding not known category.

e Place of death not known for 10 controls.

**Table 3:** Results of multivariable logistic regression model for breast cancer showing characteristics predictive of death within 30 days of diagnosis

**Characteristic**	**Odds ratio**	**95% Confidence interval**		***P*-value**
*Age group*				
<65	1.00			
65–74	2.72	1.06	6.96	0.037
75–79	6.93	2.87	16.76	<0.001
80–84	6.34	2.52	15.98	<0.001
85+	8.70	3.80	19.88	<0.001
				
*SIMD fifth*				
1-Least deprived	1.00			
2	1.47	0.86	2.51	0.154
3	1.96	1.15	3.31	0.013
4	1.17	0.69	1.99	0.563
5-Most deprived	1.05	0.61	1.80	0.861
				
*Emergency admissions within 30 days of diagnosis date*				
0	1.00			
⩾1	26.93	11.45	63.33	<0.001
				
*Microscopically verified*				
Yes	1.00			
No	6.76	4.64	9.83	<0.001
Not known	5.21	2.22	12.20	<0.001
				
*Tumour stage*				
I	1.00			
II	0.62	0.27	1.42	0.254
III	1.08	0.44	2.66	0.868
IV	5.28	2.74	10.17	<0.001
Not known	3.55	1.95	6.48	<0.001
				
*Tumour grade*				
I–II	1.00			
III	3.20	1.99	5.13	<0.001
Not known	3.20	1.99	5.13	<0.001
				
*Any treatment within 30 days of diagnosis*				
Yes	1.00			
No	4.20	3.01	5.86	<0.001

Abbreviation: SIMD=Scottish index of multiple deprivation.

The interaction term age × emergency admissions was included in the model.

**Table 4:** Characteristics of patients dying within 30 days of diagnosis of colorectal cancer in Scotland; period of diagnosis 2003–2007

	**Cases**		**Controls**		
**Characteristic**	**Number**	**%**	**Number**	**%**	***P*-value**
*Age group*					<0.001
<65	131	11.6	4602	28.9	
65–74	234	20.7	4884	30.6	
75–79	220	19.5	2793	17.5	
80–84	257	22.8	2138	13.4	
85+	287	25.4	1529	9.6	
					
*Sex*					0.002
Male	564	50.0	8721	54.7	
Female	565	50.0	7225	45.3	
					
*SIMD fifth* a					<0.001
1-Least deprived	153	13.6	3046	19.1	
2	210	18.6	3112	19.5	
3	260	23.0	3347	21.0	
4	264	23.4	3411	21.4	
5-Most deprived	242	21.4	3029	19.0	
					
*Previous inpatient bed days*					<0.001
0	338	29.9	5488	34.4	
1–7	257	22.8	5070	31.8	
8–28	269	23.8	3512	22.0	
29+	265	23.5	1876	11.8	
					
*Previous admission for COPD*					<0.001
Yes	134	11.9	831	5.2	
No	995	88.1	15 115	94.8	
					
*Previous alcohol-related admission*					<0.001
Yes	83	7.4	598	3.8	
No	1046	92.6	15 348	96.2	
					
*Emergency admissions within 30 days of diagnosis date*	<0.001				
0	212	18.8	10 108	63.4	
⩾1	917	81.2	5838	36.6	
					
*Microscopically verified*					<0.001
Yes	666	59.0	15 028	94.2	
No	441	39.1	856	5.4	
Not known	22	1.9	62	0.4	
					
*Tumour sub-site*					<0.001^b^
Proximal colon	323	28.6	4912	30.8	
Distal colon	258	22.9	4213	26.4	
Rectum/rectosigmoid	258	22.9	5533	34.7	
Not known	290	25.7	1288	8.1	
					
*Dukes' stage*					<0.001^c^
A	17	1.5	1757	11.0	
B	97	8.6	4176	26.2	
C	127	11.2	3893	24.4	
D	371	32.9	2709	17.0	
Not known	517	45.8	3411	21.4	
					
*Tumour grade*					<0.001^d^
I	8	0.7	411	2.6	
II	264	23.4	8460	53.1	
III	128	11.3	2186	13.7	
IV	8	0.7	97	0.6	
Not known	721	63.9	4792	30.1	
					
*Any treatment within 30 days of diagnosis*	<0.001				
Yes	425	37.6	6876	43.1	
No	704	62.4	9070	56.9	
					
*Underlying cause of death*					<0.001
Colorectal cancer	822	72.8	5678	77.1	
Other cancer	59	5.2	468	6.4	
Diseases of circulatory system	114	10.1	678	9.2	
Diseases of respiratory system	39	3.5	195	2.6	
Any other cause	95	8.3	345	4.7	
					
*Place of death* e					<0.001
NHS acute hospital	929	82.1	3691	50.2	
Home	105	9.2	1895	25.8	
Hospice	42	3.7	1233	16.8	
Other institution	53	4.5	530	7.2	

Abbreviations: COPD=chronic obstructive pulmonary disease; SIMD=Scottish index of multiple deprivation.

a SIMD not known for one control.

b *P*<0.001 excluding not known category.

c *P*<0.001 excluding not known category.

d *P*<0.001 excluding not known category.

e Place of death not known for 15 controls.

**Table 5:** Results of multivariable logistic regression model for colorectal cancer showing characteristics predictive of death within 30 days of diagnosis

**Characteristic**	**Odds ratio**	**95% Confidence interval**		***P*-value**
*Age group*				
<65	1.00			
65–74	2.50	1.28	4.89	0.007
75–79	5.41	2.76	10.60	<0.001
80–84	11.13	5.78	21.43	<0.001
85+	15.92	8.15	31.07	<0.001
				
*SIMD fifth*				
1-Least deprived	1.00			
2	1.22	0.97	1.55	0.095
3	1.43	1.14	1.79	0.002
4	1.34	1.07	1.69	0.01
5-Most deprived	1.16	0.92	1.46	0.204
				
*Previous inpatient bed days*				
0	1.00			
1–7	0.81	0.67	0.98	0.026
8–28	0.87	0.72	1.05	0.135
29+	0.96	0.78	1.17	0.669
				
*Previous admission for COPD*				
No	1.00			
Yes	1.46	1.17	1.84	0.001
				
*Previous alcohol-related admission*				
No	1.00			
Yes	1.59	1.19	2.12	0.002
				
*Emergency admissions within 30 days of diagnosis date*				
0	1.00			
⩾1	9.41	5.45	16.25	<0.001
				
*Microscopically verified*				
Yes	1.00			
No	8.43	4.92	14.45	<0.001
Not known	3.27	0.81	13.13	0.095
				
*Tumour sub-site*				
Proximal colon	1.00			
Distal colon	1.25	1.04	1.50	0.02
Rectum/rectosigmoid	1.11	0.92	1.35	0.272
Not known	2.19	1.80	2.67	<0.001
				
*Dukes' stage*				
A or B	1.00			
C	1.48	1.12	1.94	0.006
D	1.71	1.26	2.32	0.001
Not known	1.35	0.98	1.84	0.064
				
*Tumour grade*				
I–II	1.00			
III–IV	1.50	1.20	1.88	<0.001
Not known	1.48	1.22	1.79	<0.001
				
*Any treatment within 30 days of diagnosis*				
Yes	1.00			
No	0.17	0.08	0.38	<0.001

Abbreviations: COPD=chronic obstructive pulmonary disease; SIMD=Scottish index of multiple deprivation.

Interaction terms age × emergency admissions, age × microscopic verification, age × any treatment, and Dukes' stage × any treatment were included in the model.

**Table A1:** Table A1

**ICD-9 code**	**ICD-10 code**	**Condition**
*COPD*		
490	J40	Bronchitis, not specified as acute or chronic
	J41	Simple and mucopurulent chronic bronchitis
491	J42	Unspecified chronic bronchitis
492	J43	Emphysema
496	J44	Other COPD
		
*Alcohol-related diagnoses*		
	E244	Alcohol-induced Pseudo-Cushing's syndrome
	E512	Wernicke′s encephalopathy
291	F10	Mental and behavioural disorders due to use of alcohol
303		
	G312	Degeneration of nervous system due to alcohol
3575	G621	Alcoholic polyneuropathy
	G721	Alcoholic myopathy
4255	I426	Alcoholic cardiomyopathy
5353	K292	Alcoholic gastritis
5710	K70	Alcoholic liver disease
5711		
5712		
5713		
	K860	Alcohol-induced chronic pancreatitis
	O354	Maternal care for (suspected) damage to fetus from alcohol
7903	R780	Finding of alcohol in blood
9800	T510	Toxic effect of ethanol
9801	T511	Toxic effect of methanol
3050	T519	Toxic effect of alcohol, unspecified
9809		
E860	X45	Accidental poisoning by and exposure to alcohol
	X65	Intentional self-poisoning by and exposure to alcohol
	Y15	Poisoning by and exposure to alcohol undetermined intent
E9473	Y573	Alcohol deterrents
	Y90	Evidence of alcohol involvement determined by blood alcohol level
	Y91	Evidence of alcohol involvement determined by level of intoxication
	Z502	Alcohol rehabilitation
	Z714	Alcohol abuse counselling and surveillance
	Z721	Alcohol use

## **Appendix 1**. Diagnostic codes used to identify hospital admissions with chronic obstructive pulmonary disease (COPD) and alcohol-related diagnoses

Table A1
